# Tuberculosis prevalence in animals and humans in the Republic of Kazakhstan

**DOI:** 10.14202/vetworld.2021.2362-2370

**Published:** 2021-09-11

**Authors:** Kairat Altynbekovich Turgenbayev, Assiya Madenovna Borsynbayeva, Aleksandr A. Plazun, Rauan K. Turgenbayev

**Affiliations:** 1 Kazakh Scientific Research Veterinary Institute, 223 Raiymbek St., Almaty, 050016, Kazakhstan; 2 Research and Production Center BioVet, 191-A Karasay batyr St., 191-A, Almaty, 050008, Kazakhstan.

**Keywords:** diagnostics, epizootiology, tuberculin, tuberculosis

## Abstract

**Background and Aim:**

Tuberculosis is an infectious disease that affects humans and animals. This study aimed to review the influence of economic and organizational, veterinary, and sanitary measures on the epizootic situation of animal tuberculosis and the epidemiological situation of human tuberculosis in the Republic of Kazakhstan.

**Materials and Methods:**

The epizootic situation of cattle tuberculosis in Kazakhstan was studied based on the annual statistics of allergy testing of animals with tuberculosis by the Committee for Veterinary Control and Supervision of the Ministry of Agriculture of the Republic of Kazakhstan, according to the results of allergic, clinical, and pathological studies, including laboratory studies of biomaterials from animals that reacted to tuberculin. Tuberculinization of cattle in the country is performed twice in the spring and autumn using tuberculin purified protein derivative for mammals. In addition, mass diagnosis of human tuberculosis is conducted by radiology.

**Results:**

The authors assessed the epizootic situation of tuberculosis in cattle in Kazakhstan from 1991 to 2019. The analysis results showed that at the beginning, the epizootic situation in cattle tuberculosis was challenging. However, since 2001, there has been a noticeable decrease in tuberculosis in animals in Kazakhstan, which has had a favorable effect on the epidemic situation of human tuberculosis.

**Conclusion:**

According to the analysis results, the measures used in the Republic of Kazakhstan to prevent tuberculosis improved the well-being of livestock farms. The proper conduct by qualified veterinary specialists on allergy testing of animals and differential diagnosis of nonspecific tuberculin reactions allows establishing an accurate epizootic picture of tuberculosis. Furthermore, strict adherence to instructions on the diagnosis and prevention of tuberculosis, implementation of developed veterinary, sanitary, and organization, and economic measures can improve the epizootic picture of tuberculosis, reducing the risk of human tuberculosis.

## Introduction

Tuberculosis is a widespread infectious disease of animals and humans. In some regions of Kazakhstan, it causes significant economic damage and has great social significance. The bovine tuberculosis pathogen, recognized as a chronic zoonotic infection by the World Organization for Animal Health (Office International des epizooties; [OIE]), is *Mycobacterium bovis* [1]. However, some authors [2], based on recent phylogenomic analyses of species of the *Mycobacterium tuberculosis* complex (MTC), suggest that *M. tuberculosis* species, *Mycobacterium africanum*, *M. bovis*, *Mycobacterium caprae*, *Mycobacterium microti*, and *Mycobacterium pinnipedii*, be grouped, and they used the infraspecies term “var.,” for example, *M. tuberculosis* var. *bovis*. Tuberculosis is an infectious anthropozoonotic disease affecting mainly cattle and is the cause of 5-10% of human cases of tuberculosis, also being widespread in domestic and wild animals [3-6]. Cattle, compared with other animal species, are most susceptible to this disease [7].

Due to the existence of a scientifically grounded system of measures in Kazakhstan, there has been a tendency to improve the epizootic situation of tuberculosis. However, tuberculosis remains a complex infectious pathogen of animals. It most often chronically occurs and is characterized by several specific body changes, including the formation of tuberculosis foci in the lungs and lesions of regional lymph nodes. The chronic and asymptomatic course has led to its significant spread. Tuberculosis is most common among cattle, and its increased prevalence in humans corresponds to areas of intensive cattle breeding [8]. However, according to Donchenko *et al*. [9], the relationship between tuberculosis in cattle and humans has been weakly traced since animals can contract tuberculosis in a latent form, and the territory in which they are kept is considered tuberculosis-free.

Cattle tuberculosis on the territory of Kazakhstan was registered back in the 1930s, and for more than 70 years, the epizootic situation of this disease remained challenging. Infection manifestation was characterized by the stationarity of areas affected by tuberculosis in certain regions of Kazakhstan, the reemergence of infection in the previously rehabilitated farms, and the spread of the bovine tuberculosis pathogen among people. During the Soviet reign, party and economic bodies, by administrative measures, prohibited the reduction of animal population in farms and demanded the fulfillment of the plan for the supply of livestock products, such as milk and meat. Because of the untimely removal of sick animals and their overexposure in the herd, healthy animals were reinfected, and the infection spread. As a result, farms affected by a double infection (tuberculosis and brucellosis) were widespread. In some farms, cattle sick with tuberculosis were kept for a long time in “isolation wards,” which were not examined.

The Soviet Union, including Kazakhstan, disintegrated in 1991. The Republic of Kazakhstan proclaimed its independence and began reforming its economy, which also affected agriculture. State and collective farms were denationalized, and new private agricultural formations were created. Since 2000, in Kazakhstan, one could observe the creation of small agricultural formations, including family-operated and private farms, and a significant increase in livestock in the private sector. Organizational, economic, structural, and legal transformations aimed at creating conditions for stabilizing agricultural production. Thus, a diversified economy with various forms of ownership was formed. The main problem in preventing tuberculosis in animals was the weak economic condition of economic entities and animal owners. The diversity of agricultural formations provided for a qualitatively new approach of veterinary specialists in performing special livestock events in the personal, subsidiary, family-operated, and private farm households, which contained up to 80% of cattle. However, the measures taken to eradicate tuberculosis in farm animals did not meet the requirements, aggravating the resulting complex epizootic situation. The decline in the epizootic situation caused a complication of the epidemiological situation of tuberculosis among the population. In this regard, the Government of the Republic of Kazakhstan adopted resolution No. 839, “On urgent measures to protect the population of the Republic of Kazakhstan from tuberculosis.”

**Figure-1:**
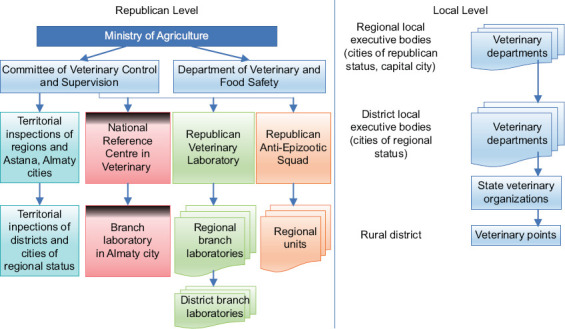
Structure of the Veterinary Service of the Republic of Kazakhstan.

In connection with the change in the culture of animal husbandry, there was a shift in emphasis on the development of methods of intravital diagnostics, means of specific prevention, and improving the systems of measures to combat tuberculosis in animals. Along with improving chronic infections at farms, the isolation of animals that reacted to tuberculin continued in tuberculosis-free agricultural entities. There was a reorganization of the structure of the veterinary service and the adoption of new veterinary legislation. Figure-1 shows the structure of the Veterinary Services of Kazakhstan, according to Article 6 of the “Veterinary Law” [10].

Diagnostic tests for tuberculosis are conducted by the veterinary service specialists of the regional offices of the Veterinary Control and Surveillance Committee twice a year, in spring, before the animals are put out to pasture and in autumn when the animals return to their stables. It is mandatory to test all cattle and, if necessary, other animal species. An intradermal allergy test with purified protein derivative (PPD)-tuberculin for mammals is used for diagnosis. If tuberculin-responsive animals are detected in a tuberculosis-free farm, they are separated from the main herd, and differential diagnostic tests are performed using a simultaneous test and diagnostic slaughter for veterinary and sanitary examination of the carcasses. If morphological changes in internal organs are detected, biomaterial samples are taken for bacteriological examination at regional divisions of the Republican Veterinary Laboratory. If tuberculin-responsive animals are detected in a diseased farm, they are recognized as infected and slaughtered within 5 days. According to official veterinary reporting, the territory of the Republic of Kazakhstan is free of animal tuberculosis. The frequency of isolation of animals with tuberculosis in Japan is 10% [11], 17% in Canada [12], and 24.5% in Russia [9]. In Germany, animals reacting to tuberculin with a skin fold thickening of 3 mm or more are considered sick and culled [13]. In addition, when such animals are identified in tuberculosis-free farms, intravital and postmortem examinations are conducted to exclude or confirm the diagnosis of tuberculosis. Furthermore, multiple examinations of cattle with intradermal allergy tests are uninformative enough (with an efficiency of 54.2%) [14]. If no infectious agents of tuberculosis are isolated from the postslaughter material of animals that reacted to tuberculin, this indicates the nonspecific nature of tuberculin reactions [15]. This causes economic damage to the development of animal husbandry due to the unjustified slaughter of outwardly healthy, often breeding, and highly productive livestock and unjustified antituberculosis veterinary and sanitary measures.

The main reason for maintaining a challenging epizootic situation in cattle tuberculosis is the resistance of mycobacteria to adverse factors and long-term survival in environmental objects [16]. In addition*, M. tuberculosis* adapts to antibiotics; thus, modern medicines have a less detrimental effect on circulating populations of mycobacteria [17].

This research aimed to study the epizootic situation of animal tuberculosis in the Republic of Kazakhstan, the effectiveness of special veterinary, organizational, and economic measures in reducing tuberculosis prevalence in animals, and sanitation of farms from the infection. Furthermore, we show how the reduction in tuberculosis prevalence in animals has affected the prevalence of human tuberculosis in the Republic of Kazakhstan. In addition, we monitor the spread of tuberculosis in the different regions of the Republic of Kazakhstan and assess the strategy to control animal tuberculosis based on their research.

## Materials and Methods

### Ethical approval

The research protocol was discussed and approved at the meeting of the local ethical committee of the Kazakh Research Veterinary Institute of the Science Committee of the Ministry of Education and Science of the Republic of Kazakhstan on August 29, 2017.

### Study period and location

The study of the epizootic situation of tuberculosis in cattle was carried out in the Republic of Kazakhstan for the period from 1991 to 2019. Experimental studies on laboratory diagnosis of tuberculosis were carried out at the Kazakh Research Veterinary Institute in Almaty.

### Methods of epizootic monitoring

Epizootic monitoring for tuberculosis of farm animals was conducted according to the “Epizootic research methods and theory of the epizootic process” [18]. The epizootic situation of tuberculosis in cattle in Kazakhstan was studied based on the materials of the official veterinary statistics of the Committee for Veterinary Control and Supervision (CVCS) of the Ministry of Agriculture of the Republic of Kazakhstan. This was published on the Ministry’s website and the results of allergic, clinical studies of cattle, clinical and pathological data, veterinary and sanitary analysis of carcasses, and laboratory studies of biomaterials from animals that reacted to tuberculin. Furthermore, when analyzing the causes of repeated cases of the disease in the previously rehabilitated farms, we considered the results of epizootic examinations conducted jointly with the veterinary service of regions, districts, and farms, under the instruction “On the measures to prevent and eliminate animal tuberculosis” [19].

### Single intradermal cervical test

The main method of *in vivo* diagnosis of animal tuberculosis is the allergy method (single intradermal cervical test). The tuberculin (PPD) for mammals produced by the BioVet Research and Production Center (the Republic of Kazakhstan) with an activity of 50,000 TU in 1 mL is used. The activity of tuberculin was harmonized with that of PPD bovine tuberculin obtained from the National Institute for Biological Standards and Control (United Kingdom). Tuberculin was injected into animals with a needleless injector intracutaneously in a dose of 0.2 mL into a preshaved and 72% ethanol-treated area of the upper third of the neck. The reaction was recorded after 72 h. A thickening of the skin fold by 3 mm or more was considered positive. The OIE recommends producing tuberculin from virulent strains of bovine mycobacteria, *M. bovis* [20]. According to some researchers [21], in tuberculin, mycobacteria retain their viability in altered forms and can cause the spread of tuberculosis during mass diagnostic studies. Based on this, there is a likelihood of artificial circulation of tuberculosis in nature [22].

### Bacteriological methods

Studies of the selected biomaterial were conducted in the bacteriology laboratory of the Kazakh Research Veterinary Institute. During the work, bacteriological methods for diagnosing tuberculosis were used with the presowing processing of diagnostic biomaterial and biological tests. For this, the material was cut into small pieces in a mortar, covered with 5% sulfuric acid solution, and left for 20 min to suppress the growth of acid-unstable microflora. Then, the acid was decanted, and the material was washed with saline and homogenized. The resulting suspension of biomaterial samples was sown on the surface of a dense nutrient Lowenstein–Jensen medium (control) and thermostated at 37°C.

### The diagnosis of tuberculosis in humans

The mass diagnosis of tuberculosis in humans is conducted by X-ray, and every working citizen of the republic must undergo a fluorography once a year. In addition, applicants, students, job applicants, and the rest of the population must be diagnosed by X-ray when they visit a health center. If the X-ray shows signs of tuberculosis, the patient is referred to a tuberculosis clinic, where X-rays, bacteriological tests on sputum samples, and polymerase chain reaction (PCR) tests are conducted.

## Results

**Figure-2:**
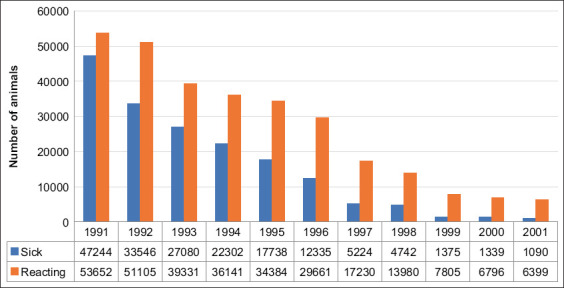
Dynamics of animals reacting to tuberculin and sick with tuberculosis from 1991 to 2001.

From 1991 to 1999, 37,670 cattle were slaughtered or died from all infectious diseases in Kazakhstan, including 21,631 animals that died of tuberculosis, amounting to 59%. According to the results of the antituberculosis measures performed in Kazakhstan for 10 years from 1991 to 2000, 290,085 animals reacting to tuberculin and 172,925 patients with cattle tuberculosis were found, which amounted to 463,010 herds (Figure-2). Animals with a positive allergic reaction in a tuberculosis-free farm were ^­^considered responsive to tuberculin, and those with characteristic changes in internal organs at slaughter, isolation of the tuberculosis pathogen in bacteriological ­examination of biomaterial, and the case ­identifying animals responsive to tuberculin in a tuberculosis-positive farm were considered sick. As a result, 553,983 herds were handed over for slaughter, considering the store cattle. Among other infectious diseases of cattle within the decade, the proportion of animals with tuberculosis was 16.6%.

Figure-2 demonstrates the dynamics of a decrease in the number of cattle reacting to and suffering from tuberculosis in Kazakhstan over 10 years. Thus, in 1991, 53,652 animals were registered as reacting to tuberculin and 47,244 animals as sick with tuberculosis in tuberculosis-free farms. In 2001, 6399 reacting animals, which is 11.9 times less, and 787 sick animals, which is 1.67 times less, were registered. In 1991, 0.8% of reacting animals was found at 388 tuberculosis-free farms. As a result, tuberculosis was confirmed in 20 farms. In 2001, at 244 tuberculosis-free farms, 0.1% of responding animals was found. Thus, tuberculosis was confirmed in 16 cases.

**Figure-3:**
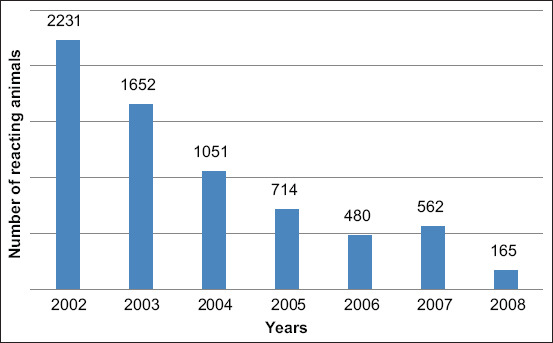
Dynamics of identification of animals reacting to tuberculin from 2002 to 2008.

**Figure-4:**
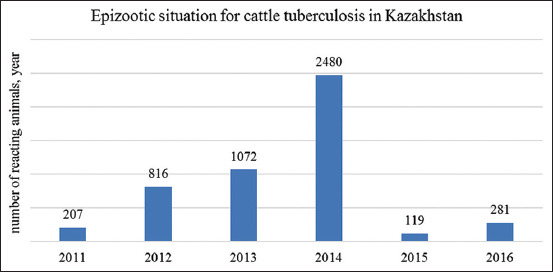
Dynamics of cattle reacting to tuberculin in Kazakhstan for 2011-2016.

Since 2002, there has been a decrease in animals reacting to tuberculin, from 2231 herds in 2002 to 165 herds in 2008 (Figure-3). This is most likely due to a reduction in the total number of cattle when those culled were sent for slaughter in the first instance, including animals that reacted to tuberculin. The epizootic data on cattle tuberculosis in Kazakhstan for 2010-2016 are shown in Figure-4. Figure-4 shows that in 2011, 207 herds reacting to tuberculin were found; in 2012, 816 animals that reacted to tuberculin were found. The diagnosis of tuberculosis was confirmed in 529 cases. The percentage of responding animals was 0.013, and those with tuberculosis were 0.009%.

In 2012, three farms were registered as affected by tuberculosis. The Ushtube-Aydin LLP located in Baktybai of the Eskeldinsky district of the Almaty region was considered to be affected by tuberculosis. Out of 440 animals studied, 54 reacting to tuberculin were found, amounting to 12.3%. Postmortem assessment of macroscopic changes, histopathology, and bacteriological research methods is the standard method for diagnosing tuberculosis in animals [20]. Therefore, we conducted the control and diagnostic slaughter of five animals from the responding ones; veterinary and sanitary examinations established the diagnosis of tuberculosis, and the cultures of mycobacteria were isolated from the biomaterial samples in the Institute’s laboratory, which, according to their cultural and morphological properties, were assigned to the bovine species. As a result of the studies, all animals that reacted to tuberculin were recognized as having tuberculosis.

**Figure-5:**
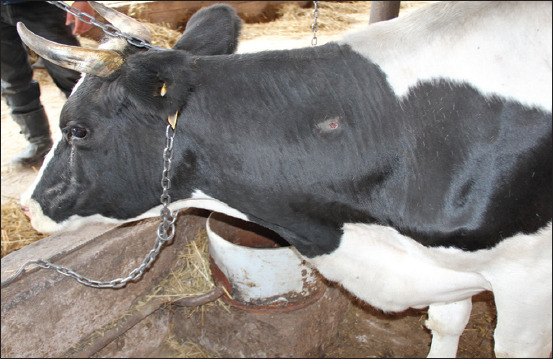
Allergic reaction in a cow in the Shokan and Co. LLP to the injection of tuberculin with skin necrosis at the injection site.

**Figure-6:**
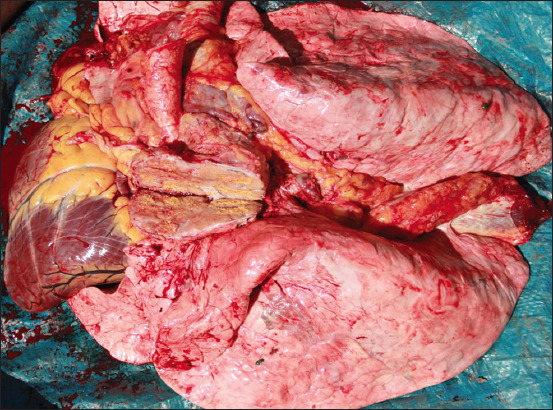
Changes in the mediastinal lymph node characteristic of tuberculosis in a cow that reacted to tuberculin at the Shokan and Co. LLP.

In the Bazarkhanov LLP, located in Ushtoba, the Karatal district of the Almaty region, based on a veterinary and sanitary analysis of the carcasses, tuberculosis was identified in 11 animals, and we isolated *M. tuberculosis* cultures from biomaterial samples. In the Shokan and Co. LLP, located in Shokan, Saryozek district, Almaty region, during a planned allergy study, 124 animals that reacted to tuberculin were found (Figure-5). In addition, during the control and diagnostic slaughter of two cows, we found tuberculous changes by veterinary and sanitary examination of the carcasses (Figure-6). Mycobacteria cultures were identified from the selected biomaterial samples, which, according to their cultural, morphological, and biological properties, were assigned to the bovine species.

**Figure-7:**
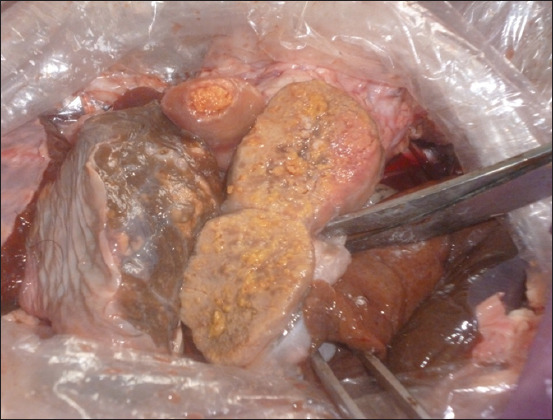
Changes typical for tuberculosis in the pharyngeal lymph node of a cow from the Prirechenskoye LLP.

In 2013, 6,897,895 herds were initially tested for tuberculosis in Kazakhstan. Simultaneously, 1072 animals (0.016%) reacting to tuberculin were found, and in 719 heads (0.01%), the diagnosis of tuberculosis was confirmed during slaughter. A high percentage of morbidity in cattle was noted in the Kostanay region (0.2%). The Prirechenskoye LLP, Denisov district, Kostanay region, where 717 cows with a positive reaction were found at one farm, was declared affected by tuberculosis. During the diagnostic slaughter of animals that reacted to tuberculin, visible tuberculous changes in the internal organs and lymph nodes were established (Figure-7).

Figure-7 shows changes in the lymph node characteristic of tuberculosis, with a pronounced purulent-caseous lesion. We studied samples of biomaterials (lymph nodes, liver, lungs, and kidneys) selected from 24 slaughtered animals microbiologically. The inoculum was processed in the Institute’s laboratory, followed by sowing on the Lowenstein–Jensen medium. As a result, mycobacteria cultures were isolated from each sample and assigned to the bovine species by their cultural, morphological, and biological properties.

In the Karaganda region, in the Shet district, at the Sulu Madine farm, 64 herds that reacted to the PPD tuberculin for mammals were found. At the slaughter of the reacting animals, a veterinary and sanitary analysis of carcasses confirmed the diagnosis of tuberculosis. As a result, tuberculosis prevalence in animals on average in the rural district equaled 0.015%. In the Nuftiyeva private enterprise of the Almaty region, during a planned allergic study of 78 cows, 17 reacting animals were found, during the slaughter of which we also found tuberculosis in the internal organs by veterinary and sanitary examination.

In 2014, 1690 animals that reacted to tuberculin were found in the Kostanay region. In the Karaganda region, 790 herds responded to tuberculin, including in the Abai district, the Yubileiny rural district, the Mayburnak winter farm, and the Ata family-operated farm. The diagnosis of tuberculosis was confirmed by veterinary and sanitary examination of carcasses during the slaughter of two reacting animals and bacteriological examination. In addition, cultures of *M. tuberculosis* were isolated from samples of the selected biomaterial.

In 2015, cattle tuberculosis was registered in the Karaganda region, Bukhar-Zhyrau district, Dubovsky rural district in the village of Alabas, and the Arslan family-operated farm. During the selective control and diagnostic animal slaughter and veterinary and sanitary analysis of carcasses, tuberculosis was diagnosed, and 24 herds recognized as sick were sent for slaughter.

In the Almaty region, an intradermal tuberculin test was conducted on 2990 herds, and 95 (3.1%) animals that reacted to tuberculin were found. In addition, 58 herds were subjected to diagnostic slaughter. Twenty-six animal carcasses were subjected to veterinary and sanitary examination, and tuberculosis was confirmed in 100% of cases. Simultaneously, a generalized form of tuberculosis was established in five animals, and widespread tuberculosis (lymph nodes, lungs, and liver) was noted in seven animals. In five cows, specific tuberculous lesions were found in the mesenteric lymph nodes. The bacteriological method was used to study biomaterial from seven cows. In all cases, a culture of bovine mycobacteria was isolated. Furthermore, four cows that reacted to tuberculin were found at a private farmstead, and when they were slaughtered, the diagnosis of tuberculosis was confirmed by veterinary and sanitary examinations.

In 2016, in the Karaganda region in the Karkaralinsky district of the Besobinsky rural district, 281 animals that reacted to tuberculin were found in Abai during an allergy study. During the commission control and diagnostic slaughter of five cows, the veterinary and sanitary examination established the diagnosis of tuberculosis. All animals that responded to tuberculin were sick with tuberculosis and sent to meat processing institutions for slaughter. There was also a case of tuberculosis in cattle at a personal farmstead in Zhezkazgan, Karaganda region. During the veterinary and sanitary examination of the carcass, tuberculosis was established in one herd.

Recently, much work has been done in Kazakhstan to prevent tuberculosis in tuberculosis-free farms and improve the health of economic entities affected by tuberculosis. As a result, there is a shift in emphasis on developing methods of intravital diagnostics, means of specific prevention, and improving systems of measures to combat tuberculosis in animals. The CVCS of MA, employing local veterinary workers, annually conducts a double allergic study of the livestock, which as of 2020 amounted to 8,800,000 herds. According to the veterinary reports of the CVCS, no animals reacting to tuberculin were found in Kazakhstan. Presently, Kazakhstan is considered free from animal tuberculosis.

According to the chief state sanitary doctor in the Republic of Kazakhstan, there is a stable epidemiological situation concerning human tuberculosis. Over the past 8-9 years, about 4000 people were diagnosed with tuberculosis yearly. The diagnosis was confirmed by radiological and laboratory tests (PCR and bacteriological method). In 25% of these people, cultures of mycobacteria of multidrug-resistant tuberculosis were detected. Over the past 3 years, the prevalence rate of tuberculosis in people in Kazakhstan has decreased by 1.1 times (from 52.7 in 2016 to 48.2/100,000 people in 2018) and the mortality rate by 1.4 times (from 3.4 to 2.4/100,000 people). In 2019, the prevalence rate of tuberculosis was 34.7, and the mortality rate was 1.6/100,000 people. Thus, the yearly rate of decrease in morbidity and mortality is 7-8%. Effective diagnosis and treatment of tuberculosis have saved the lives of more than 53,000 people who had contracted tuberculosis over the past 3 years.

**Figure-8:**
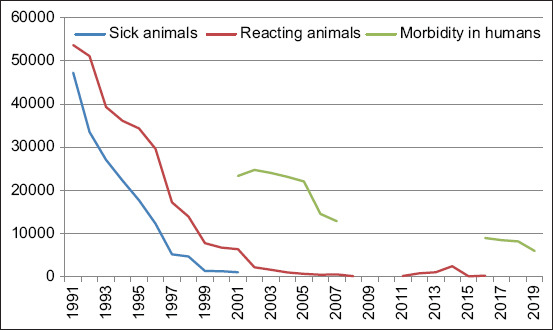
Dynamics of tuberculosis morbidity in humans and animals in Kazakhstan.

The widespread observance of the requirements in livestock farms is described in detail in the instructions for the diagnosis and prevention of tuberculosis. Furthermore, strict implementation of the developed rules allowed the reduction of tuberculosis prevalence in animals and humans. These measurements positively impacted the epizootic situation in the republic, which ultimately decreased the prevalence of tuberculosis in people in Kazakhstan (Figure-8).

## Discussion

The epizootic situation in cattle tuberculosis in Kazakhstan has significantly improved. Among the ecotypes grouped within the MTC, *M. bovis* and *M. caprae* are recognized as the most relevant causative agents of tuberculosis in animal husbandry [23-25]. The disease is characterized by the formation of granulomatous foci localized in the lymph nodes or several internal organs, mainly affecting the respiratory, digestive, and excretory systems. The anatomical localization of lesions is usually associated with transmission routes [26,27].

According to the veterinary reports, in 2011, 201 herds responding to tuberculin were found. In 2012, out of 12,369,722 animals studied, 816 animals reacting to tuberculin were found. As a result, the diagnosis of tuberculosis was confirmed at three places affected by tuberculosis in 529 herds. In 2013, 6,897,895 herds were tested for tuberculosis. Simultaneously, 1072 animals reacting to tuberculin were found. In two places affected by tuberculosis, the diagnosis of tuberculosis was confirmed in 719 herds during slaughter. In 2014, 1690 animals that reacted to tuberculin were found in the Kostanay region. In the Karaganda region, 790 herds of cattle that reacted to tuberculin were found, including in the Abay region in the Ata family-operated farm. The diagnosis of tuberculosis was confirmed when the reactive animals were slaughtered. In 2015, cattle tuberculosis was registered in the Karaganda region, the Bukhar-Zhyrau region in the village of Alabas, and the Arslan family-operated farm.

During the slaughter of the reacting animals, tuberculosis was confirmed, and 24 herds recognized as sick were sent for slaughter. In the Almaty region, 2990 herds were examined, and 95 (3.1%) animals that reacted to tuberculin were found. Furthermore, 58 herds were subjected to diagnostic slaughter, and 26 animal carcasses were subjected to veterinary and sanitary examination; tuberculosis was confirmed in 100% of cases. In 2016, in the Karaganda region in the Karkaralinsky district in the Abai procuring center, animals that reacted to tuberculin were found during an allergy study. During the slaughter, the animals were diagnosed with tuberculosis. Two hundred eighty-one herds were sick with tuberculosis and sent to meat processing establishments for slaughter. These results were expected because airborne transmission is the most common route of infection in cattle, resulting in damage to the respiratory tract and associated lymph nodes, and then oral transmission through ingestion of bacilli, resulting in damage to the digestive tract and associated lymph nodes. Furthermore, generalized systemic infection is associated with a terminal illness, which is rare due to periodic *in vivo* testing of cattle, reducing the prevalence of disseminated tuberculosis [28].

Tuberculosis is an anthropozoonotic infection. The tuberculosis pathogen circulation in nature and its impact on human and animal morbidity in the Republic of Kazakhstan were studied in the last century at the Tuberculosis Institute of the Ministry of Health in Almaty under the direction of Blagodarny [29]. Convincing data on the circulation of mycobacteria from humans to animals and vice versa were obtained. Recently, research in this direction has been considered irrelevant, and such studies have not been conducted. Much has been said about the natural foci of tuberculosis, and it is impossible to eradicate the pathogen from the external environment due to its long-term survival. Some researchers [30] have observed cyclicity in the development of the tuberculosis epizootic process and attributed this to solar activities.

In this work, a correlation between the reduction in the prevalence of tuberculosis in animals and its impact on the prevalence of tuberculosis in humans in the country has been established. Planned allergy testing of animals for tuberculosis, timely culling of compromised animals that react to tuberculin, careful veterinary and sanitary control of livestock products, and restrictions on the delivery of livestock products to consumers from sick animals reduce the risks of tuberculosis people. Implementing special veterinary and sanitary measures to control tuberculosis in Kazakhstan allowed the recovery of livestock farms from this chronic infection. Thus, it is necessary to continue to carry out the measures stipulated in the instruction “On measures to prevent and eliminate tuberculosis in animals” to maintain the achieved status.

## Conclusion

The epizootic situation of bovine tuberculosis in the Republic of Kazakhstan was studied. According to the analysis results, the measures used in the Republic of Kazakhstan to prevent tuberculosis have improved the well-being of livestock farms. Appropriate allergy testing by qualified veterinary professionals and differential diagnosis of nonspecific tuberculin reactions can establish the true epizootic picture regarding tuberculosis. Strict adherence to the instructions for diagnosing and preventing tuberculosis, implementing developed veterinary and sanitary, and organizational and economic measures can improve the epizootic of tuberculosis, decreasing the risk of tuberculosis prevalence in humans.

## Authors’ Contributions

KAT: Conception and design, acquisition of data, analysis and interpretation of data, and drafted and revised the manuscript critically. AMB: Conception and design, drafted and revised the manuscript critically. AAP: Acquisition of data, and analysis and interpretation of data. RKT: Conception and design, acquisition of data, and analysis and interpretation of data. All authors read and approved the final manuscript.
